# Influenza-Associated Mortality in Georgia (2009–2011)

**DOI:** 10.1155/2012/480763

**Published:** 2012-08-15

**Authors:** Maia Butsashvili, George Kandelaki, Khatuna Zakhashvili, Ana Machablishvili, Nata Avaliani

**Affiliations:** National Center for Disease Control and Public Health, 9 Asatiani Street 0177 Tbilisi, Georgia

## Abstract

We analyzed data from NCDCPH Georgia where samples from outpatients with influenza-like illness (ILI) and inpatients with severe acute respiratory syndrome (SARI) are referred for testing on influenza virus using PCR analysis. During 2009-2010 and 2010-2011 influenza pandemics total number of the laboratory-confirmed influenza cases were 1286 with 33 deaths (all of them influenza type A) and 1203 (51.4% type A) with 44 deaths, respectively. At least one underlying medical condition was reported in 70.7% (for pandemic influenza strain) and 96% (for influenza type B) of deaths. Predominating preexisting condition was coronary heart disease.

## 1. Background

Pandemic and seasonal influenza result in significant morbidity, increase in hospitalization rate and mortality. Even though influenza is usually mild and self-limited disease, among certain population groups, such as elderly people, very young children and patients with different underlying medical conditions (diabetes, cardiovascular and pulmonary comorbidities, and other chronic diseases), it may pose a serious risk with potential complications and death [1]. 

2009 influenza pandemic involved the new strain of H1N1 virus. The pandemic had started in April 2009 in Mexico and spread worldwide. During 2010-2011 epidemic, according to the WHO report from countries of European Union, about 90% of subtyped influenza viruses from the hospitalized cases were pandemic strain of A (H1N1), 1%-A(H3N2) and 10%-influenza B viruses [2]. 

Unlike the seasonal influenza, during the 2009 pandemic the higher attack rates were documented among young adults compared to persons older than 60 [3, 4]. For both influenza seasons, neuraminidase inhibitors were recommended for treatment of pregnant women, children under two years, patients with severe, progressive disease, and for those having underlying chronic diseases.

The objective of the study was to estimate mortality and underlying medical conditions among patients with influenza during 2009-2010 and 2010-2011 seasons in Georgia. The country started influenza surveillance from 2007 when Georgian National Center for Disease Control and Public Health (NCDCPH) became the National Influenza Center, a part of WHO Global Influenza Surveillance Network.

## 2. Methods

We analyzed the data from NCDCPH where the samples from outpatients with influenza-like illness (ILI) and inpatients with severe acute respiratory syndrome (SARI) from sentinel sites throughout the country are referred to be tested for influenza virus. The following case definitions were used: ILI was defined as acute onset of fever >38°C, and cough and/or sore throat in the absence of other diagnosis. SARI was defined as acute onset of fever >38°C, cough and/or sore throat and signs of respiratory distress requiring hospitalization. For ILI the time interval between sampling and onset of symptoms was defined as <72 hours. Each primary health care provider was responsible to collect specimens 2 times per week from all the patients seeking for medical assistance that day and who satisfied case definition.

Combined nasopharyngeal/oropharyngeal swabs were taken for testing. Endotracheal aspirates were used for intubated patients. Specimens were placed into appropriate transport media and delivered to the laboratory using cold packs. The laboratory investigations were performed by PCR analysis. Influenza virus RNA was extracted from 140 *μ*L of each clinical specimen using QIAmp viral RNA mini kit (Qiagen, Germany) according to the manufacturer's instructions. Viral RNA detection was performed using primers and probes provided by US Centers for Disease Control and Prevention. CDC protocol for real-time RasT-PCR was used for Roche light cycle 2.0 [5]. 

For the patients with lethal outcome detailed demographic and medical information was collected by chart review using data collection instrument. Collected medical data included underlying chronic disease, clinical diagnosis, and treatment and vaccination history.

## 3. Results

### 3.1. Influenza Strains

During the 2009-2010 influenza pandemic the total number of the laboratory-confirmed influenza cases was 1286 (Table 1). All of them were identified as influenza type A (H1N1). The majority of registered cases were within the age groups of 5–14 and 15–29. During the 2010-2011 influenza epidemic 1203 cases were confirmed. The predominating virus was still H1N1 (51.4% of all registered cases) with 48.5% typed as influenza B. Only one influenza case was related to H3N2. The laboratory confirmation rates for patients with ILI were 12.6% and 25% for 2009-2010 and 2010-2011 seasons, respectively. Among patients with SARI laboratory confirmed influenza was diagnosed in 26% in 2009-2010 and 31% in 2010-2011.

### 3.2. Influenza-Associated Mortality

The overall mortality among patients with laboratory confirmed influenza cases was 2.56% and 3.65% during 2009-2010 and 2010-2011 seasons, respectively. Among patients with influenza type A the proportion of lethal outcome was 3.25% and 2.57%—for type B.

The mean age of patients with influenza-associated deaths was 46.7 for type A influenza virus and 62.4 for type B virus (*P* = 0.012).

The absolute number of deaths due to influenza A was highest in the age group of 30–64 years (51.5% and 65.5%, resp., out of total number of deaths in 2009-10 and 2010-11), but the case fatality ratio was highest in the age group 65+ during both seasons—11.1% and 14.7%, for 2009-2010 and 2010-2011, respectively. For influenza type B absolute number of deaths as well as case fatality ratio was highest in the age group of 65 and older (67% out of total number of deaths with 18.8% case fatality ratio).

### 3.3. Distribution of Underlying Medical Conditions among Patients with Lethal Outcome

Total numbers of influenza-related deaths were 33 and 44 during 2009-2010 and 2010-2011 seasons, respectively (Table 1). At least one underlying medical condition was reported in 70.7% of deaths related to pandemic influenza strain and 96% of deaths related to influenza type B (Table 2). 19% of patients with influenza type A and 68% of patients with influenza type B had coronary heart disease. Other predominating preexisting conditions among lethal cases with pandemic influenza strain were pregnancy (8.6%), diabetes (13.8%), obesity (10.3%), and neurological disorders (8.6%). 21.7% of deaths occurred among previously healthy individuals. Although the majority of individuals with lethal outcome (83%) were treated with neuraminidase inhibitors, none of them had received antiviral therapy within the first 48 hours of illness. None of the patients with lethal outcome were vaccinated against influenza.

## 4. Discussion

Mortality data of the two influenza seasons demonstrate that younger patients were at risk for pandemic influenza A (H1N1) associated death compared to seasonal influenza B where most of the fatal cases were observed among elderly people. The finding is consistent with the data on pandemic influenza associated death from other countries, reporting that about 90% of death cases were registered among those younger than 65 [6]. 

The majority of lethal cases occurred among patients with underlying medical conditions (only 21.7% of death among previously healthy people). Numerous studies report the similar finding of higher risk for influenza associated complications and hospitalizations among those with different chronic diseases [7, 8]. The US CDC clinical case series among patients hospitalized with A(H1N1) infection showed that 67% of patients had underlying medical condition [9]. All these emphasize the public health importance of influenza vaccination among persons from high risk groups (such as pregnant women and patients with chronic diseases). As our study showed, none of the patients with influenza associated deaths were vaccinated. Influenza vaccine is available at the vaccination centers throughout the country, but it is underutilized and only small proportion of the population is usually vaccinated. No systematic approach is used to vaccinate high risk groups and health care workers. Besides, in all fatal cases the institution of antiviral therapy was delayed beyond the 48 hour threshold, possibly contributing to the grim outcome. Early antiviral treatment when indicated should be advocated among primary care physicians to promptly initiate the therapy in order to avoid complications and reduce the lethal outcomes.

## Figures and Tables

**Table 1:** Laboratory-confirmed and lethal cases of influenza in Georgia, 2009–2011.

	2009-2010		2010-2011			
	A (H1) (*N*, %)		A (H1) (*N*, %)		B	
Age group (years)	Total cases	Lethal cases	Total cases	Lethal cases	Total cases	Lethal cases
0–4	210 (16.3)	0 (0)	150 (24.2)	1 (3.4)	187 (32.0)	0 (0)
5–14	410 (31.8)	1 (3)	91 (14.7)	1 (3.4)	139 (23.8)	0 (0)
15–29	404 (31.4)	13 (39)	189 (30.5)	3 (10.3)	95 (16.3)	0 (0)
30–54	199 (15.4)	17 (51.5)	121 (19.5)	19 (65.5)	55 (9.4)	2 (13.3)
55–64	29 (2.25)	0	31 (5)	0	45 (7.7)	3 (20)
65+	18 (1.4)	2 (6)	34 (5.5)	5 (17.2)	53 (9.0)	10 (67)
Age not registered	16 (1.2)	0	3 (0.48)	0	10 (1.7)	0

Total	1286	33	619	29	584	15

**Table 2:** Underlying medical conditions among patients with lethal outcome, by influenza virus type, Georgia 2009–2011.

	Type A (*N* = 58)	Type B (*N* = 27)
Underlying disease	*N* (%)	*N* (%)
Pregnancy	5 (8.6)	2 (8.0)
Coronary heart disease	11 (19.0)	17 (68.0)
Diabetes	8 (13.8)	0 (0)
Pulmonary diseases	5 (8.6)	3 (12.0)
Obesity	6 (10.3)	1 (4.0)
Neurological disorders	5 (8.6)	1 (4.0)
Chronic liver diseases	4 (6.9)	2 (8.0)
Lupus	1 (1.7)	1 (4.0)
Anemia	2 (3.4)	0 (0)
Chronic kidney diseases	2 (3.4)	0 (0)
Leukemia	1 (1.7)	0 (0)
Down syndrome	1 (1.7)	0 (0)
Alcoholism	0 (0)	1 (4.0)
Chest abnormality	1 (1.7)	0 (0)
Other comorbidities	7 (12%)	0 (0)
